# What Determine the Corporate Tax Rates During the COVID-19? Evidence From 113 Countries

**DOI:** 10.3389/fpubh.2021.816561

**Published:** 2022-01-04

**Authors:** Ronghua Li, Zhenhui Li, Lin Guo

**Affiliations:** ^1^School of Business, Henan Normal University, Xinxiang, China; ^2^Soft Science Research Base for Industrial Transformation and Upgrading, Henan Normal University, Xinxiang, China; ^3^School of Economics and Management, Communication University of China, Beijing, China; ^4^School of Economics, Tianjin University of Commerce, Tianjin, China

**Keywords:** COVID-19, COVID-19-related uncertainty, World Pandemic Uncertainty Index, corporate tax rates, machine learning estimators

## Abstract

Fiscal policy implications become an important tool to soften the negative consequences of the COVID-19 pandemic. Given this backdrop, this paper analyses the drivers of corporate tax rates during the COVID-19 pandemic (i.e., in 2020 and 2021). The results from 113 advanced and developing economies show that a higher level of the COVID-19-related uncertainty is positively associated with the corporate tax rates. Similarly, the country size (measured by total population) increases the corporate tax rates. Per capita income is negatively related to the corporate tax rates, but this evidence is insufficient to consider different estimation techniques. The paper also discusses potential fiscal policy implications for the driving mechanism of corporate tax rates for the post-COVID-19 era.

## Introduction

The COVID-19 pandemic has significantly affected economic indicators and societies (1). The pandemic has also created various problems in the business environment. Therefore, policymakers have put forward various measures to slow down the spread of the virus, and they have closed down public areas, restaurants, and schools (2). Most countries have implemented lockdown policies to change the spread of the COVID-19.

Notably, lockdown and other closure policies have increased the level of economic uncertainty related to the pandemic. According to Altig et al. (3), Baker et al. (4, 5), the COVID-19 related uncertainty has provided major economies. Besides, the COVID-19 related uncertainty has created both demand and supply shocks (6, 7). Policymakers have implemented fiscal policy (8, 9), income policy (10), monetary policy (11), and tax policy (12) implications. These implications are also supported by stimulus packages to promote business investments and household consumption (13, 14). These stimulus packages have created cash crunch and fiscal deficit problems in some countries (15). Therefore, countries have implemented different taxes, government debts, and other fiscal operations. The fiscal deficits have also raised the interest on corporate tax rates, providing minimum corporate tax rates globally (16).

Given this backdrop, this paper aims to examine the drivers of corporate tax rates during the COVID-19 pandemic era of 2020 and 2021. Several papers show that uncertainties significantly affect the corporate tax rates [see, e.g., (17, 18)]. Previous papers also analyse the determinants of the tax policies during the COVID-19 era. For instance, Bredemeier et al. (19) illustrate that reducing labor taxes can be an effective policy tool to promote employment in the United States during the COVID-19 era. Clemens and Veuger (20) observe that the COVID-19 pandemic has caused a significant reduction in income tax collections in the United States.

At this stage, we analyse the drivers of corporate tax rates during the COVID-19 pandemic. Previous papers have generally focused on specific tax measures in developed economies, such as the United States. For this purpose, we use the sample of 113 developed and developing economies in 2020 and 2021. To the best of our knowledge, this is the first paper in the empirical literature to examine the determinants of corporate tax rates across the countries during the COVID-19 era. We find that a higher level of the COVID-19-related uncertainty is positively associated with the corporate tax rates. Similarly, the country size (measured by total population) increases the corporate tax rates. Per capita income is negatively related to the corporate tax rates, but the evidence is insufficient to consider different estimation techniques.

The remaining parts of the study are structured as follows. Section Empirical Model, Econometric Procedures, and Data provides the details of the empirical model, the econometric procedures, and the data. Section Empirical Findings reports the empirical findings, and section Conclusion concludes.

## Empirical Model, Econometric Procedures, and Data

This paper estimates the following model in 113 advanced- and developing economies^1^ in 2020 and 2021:


(1) $$\begin{array}{l} {CTR_{i,t}=\alpha_{0}+\alpha_{1}X}_{i}+\mu_{i}\;\left(1\right) \end{array}$$

In Equation (1), *CTR*_*i, t*_ is the corporate tax rates in 2020 and 2021, *X*_*i*_ is the vector for independent variables, such as the per capita income, total population, and the World Pandemic Uncertainty Index, *i* captures the country, and μ_*i*_ represents a random error term.

We consider two econometric methods of cross-sectional data to estimate the Equation (1) for 113 countries. Country selection is based on data availability. The first is the Ordinary Least Squares (OLS) method, and the second is the Kernel-Based Regularized Least Squares (KRLS) method introduced by Hainmueller and Hazlett (21, 22).

The dependent variable is the corporate tax rates, which are the rate in 2020 and 2021, and the related data are accessed from the source of KPMG (23). We also include three independent variables at the cross-country level. Specifically, we include the per capita GDP (based on the constant $ prices) and total population in millions. These data are downloaded from the World Development Indicators dataset of the World Bank (24). In addition, we include the World Pandemic Uncertainty Index (WPUI). The WPUI measure was introduced by Ahir et al. (25), and we downloaded the data from their website (https://worlduncertaintyindex.com/data/). The WPUI measure counts the number of articles in Economist Intelligence Unit (EIU) country reports related to uncertainty due to global pandemic (the COVID-19 in our case) in different countries. Note that a higher WPUI reflects a higher uncertainty related to the global pandemic. We consider the WPUI measure in the natural logarithmic form. Note that the WPUI in 2021 is the average of the values in 2021Q1 and 2021Q2.

On the other hand, we summarize descriptive statistics (i.e., mean, standard deviation, minimum, and maximum values) in Table 1 for five indicators in the sample for 113 countries.

**Table 1:** Descriptive statistics.

**Variable**	**Definition**	**Label**	**Mean**	**Std. Dev**.	**Min**.	**Max**.	**Obs**.
Corporate Tax Rates (2021)	Per Cent	CTR2021	23.86	7.149	7.500	55.00	113
Corporate Tax Rates (2020)	Per Cent	CTR2020	24.02	7.214	7.500	55.00	113
Per Capita GDP (PPP USD)	Natural Logarithm	LnGDPC	9.438	1.117	6.554	11.67	113
Total Population (Millions)	Natural Logarithm	LnPOP	16.75	1.403	14.45	21.08	113
World Pandemic Uncertainty (Index)	Natural Logarithm	LnWPUI	5.699	0.170	5.236	6.242	113

We also report the pairwise correlations in Table 2.

**Table 2:** Correlation matrix.

**Variable**	**CTR2021**	**CTR2020**	**LnGDPC**	**LnPOP**	**LnWPUI**
CTR2021	1.000	–	–	–	–
CTR2020	0.939	1.000	–	–	–
LnGDPC	−0.260	−0.275	1.000	–	–
LnPOP	0.353	0.355	−0.169	1.000	–
LnWPUI	0.274	0.323	−0.403	0.307	1.000

Table 2 indicates the positive correlation between corporate tax rates in 2020 and 2021, the total population, and the WPUI. There is a negative correlation between corporate tax rates and per capita GDP. The WPUI is negatively correlated with the per capita GDP, but the WPUI and total population are positive. The correlation is negative between per capita GDP and population.

## Empirical Findings

Table 3 reports the findings of the cross-sectional OLS estimations for the corporate tax rates during the COVID-19 pandemic in 113 countries.

**Table 3:** OLS Results: Corporate Tax Rates during the COVID-19 pandemic.

**Variable**	**CTR (2020) (1)**	**CTR (2020) (2)**	**CTR (2020) (3)**	**CTR (2021) (4)**	**CTR (2021) (5)**	**CTR (2021) (6)**
LnGDPC	−1.779^***^ (0.589)	−1.431^***^ (0.567)	−1.020^*^ (0.606)	−1.667^***^ (0.586)	−1.319^**^ (0.565)	−1.040^*^ (0.608)
LnPOP	–	1.630^***^ (0.451)	1.409^***^ (0.463)	–	1.622^***^ (0.449)	1.471^***^ (0.465)
LnWPUI	–	–	7.421^*^ (4.111)	–	–	5.045^**^ (2.124)
Countries	113	113	113	113	113	113
R-squared (Adjusted)	0.067	0.158	0.197	0.059	0.151	0.155

*The dependent variable is the Corporate Tax Rates in 2020 (left columns) and the Corporate Tax Rates in 2021 (right columns). Constant Term is included, and the standard errors are reported in parentheses*.

*** *p < 0.01*,

** *p < 0.05, and*

* *p < 0.10*.

The columns from (1) to (3) report the results in 2020, and the columns from (4) and (6) provide the findings in 2021. Columns (1) and (4) merely include the log per capita income (LnGDPC). Columns (2) and (5) both include the log per capita income (LnGDPC) and the log total population (LnPOP). Columns (3) and (6) include the log per capita income (LnGDPC), the log total population (LnPOP), and the log World Pandemic Uncertainty Index (LnWPUI).

The findings show that LnGDPC is negatively related to the corporate tax rates and all coefficients are statistically significant at the 10% level at least. The coefficients change between −1.02 and −1.78 and meaning that a 1% increase in the per capita GDP leads to more than a 1%-point decrease in the corporate tax rates. In addition, LnPOP increases the corporate tax rates, and all coefficients are statistically significant at the 1% level. The coefficients change between 1.41 and 1.63, implying that a 1% rise in the total population yields more than a 1%-point increase in the corporate tax rates. Finally, LnWPUI is positively associated with the corporate tax rates, and all coefficients are statistically significant at the 10% level at least. The coefficients change between 5.04 and 7.42, meaning that a 1% increase in the World Pandemic Uncertainty Index leads to a more than 1%-point increase in corporate tax rates.

Furthermore, Table 4 provides the results of the cross-sectional KRLS estimations for the corporate tax rates during the COVID-19 pandemic in 113 countries.

**Table 4:** KRLS results: corporate tax rates during the COVID-19 pandemic.

**Variable**	**CTR (2020) (1)**	**CTR (2020) (2)**	**CTR (2020) (3)**	**CTR (2021) (4)**	**CTR (2021) (5)**	**CTR (2021) (6)**
LnGDPC	−0.562^*^ (0.293)	−0.466 (0.301)	−0.036 (0.375)	−0.525^*^ (0.291)	−0.420 (0.298)	−0.142 (0.325)
LnPOP	–	0.883^***^ (0.240)	0.984^***^ (0.280)	–	0.873^***^ (0.238)	0.892^***^ (0.249)
LnWPUI	–	–	6.251^**^ (2.521)	–	–	3.755^*^ (2.174)
Countries	113	113	113	113	113	113
R-squared (Adjusted)	0.062	0.161	0.286	0.059	0.162	0.227

*The dependent variable is the Corporate Tax Rates in 2020 (left columns) and the Corporate Tax Rates in 2021 (right columns). Constant Term is included, and the standard errors are reported in parentheses*.

*** *p < 0.01*,

** *p < 0.05, and*

* *p < 0.10*.

The columns from (1) to (3) provide the findings in 2020, and the columns from (4) and (6) report the results in 2021. Columns (1) and (4) only consider the log per capita income (LnGDPC). Columns (2) and (5) both include the log per capita income (LnGDPC) and the log total population (LnPOP). Columns (3) and (6) include the log per capita income (LnGDPC), the log total population (LnPOP), and the log World Pandemic Uncertainty Index (LnWPUI).

The results indicate that LnGDPC is negatively associated with corporate tax rates, but some coefficients are statistically insignificant. The coefficients change between −0.04 and −0.56 and meaning that a 1% increase in the per capita GDP leads to a less than a 1%-point reduction in the corporate tax rates. In addition, LnPOP increases the corporate tax rates, and all coefficients are statistically significant at the 1% level. The coefficients change between 0.87 and 0.99, implying that a 1% rise in the total population yields less than a 1%-point increase in the corporate tax rates. Finally, LnWPUI increases the corporate tax rates, and all coefficients are statistically significant at the 10% level at least. The coefficients change between 3.75 and 6.25, meaning that a 1% increase in the World Pandemic Uncertainty Index causes more than a 1%-point increase in corporate tax rates.

Overall, we observe that a higher log WPUI is positively related to the corporate tax rates. Similarly, the log total population increases the corporate tax rates. The per capita GDP is negatively associated with the corporate tax rates, but this result is not robust to utilize different estimation techniques, such as the OLS and the KRLS.

## Conclusion

In this paper, we analyzed the determinants of the corporate tax rates during the COVID-19 pandemic. For this purpose, we focused on the data for 2020 and 2021 in 113 developed and developing economies. We found that a higher level of the COVID-19-related uncertainty, which the World Pandemic Uncertainty Index measures, is positively related to the corporate tax rates. Similarly, the country size, measured by the log of the total population in millions, increases corporate tax rates. However, the per capita GDP is negatively associated with the corporate tax rates, but this result is not robust to utilize different estimation techniques. Therefore, we concluded that the COVID-19-related uncertainty and the country size were the main driving mechanisms of corporate tax rates during the COVID-19 era. It seems that COVID-19-related uncertainty shocks have increased the fiscal costs, and governments have increased the corporate tax rates to finance the fiscal cost due to the pandemic. In addition, the spread ratio of the COVID-19 is generally higher in large countries than in small countries; therefore, the pandemic's economic costs should be higher. Therefore, the country size can be positively related to the corporate tax rate increases, and the evidence aligns with theoretical backgrounds. In addition, we observe that the change in corporate tax rates is not significantly associated with the per capita GDP.

At this stage, it is important to note that our results are limited by considering the corporate tax rates. Therefore, future papers should focus on the changes in other tax measures, such as the indirect tax rates and the individual income tax rates in developed and developing economies. Given that per capita income is not the main driver of corporate tax rate change, future papers can analyse determinants of tax rates changes in natural resource-abundant and natural resources scarce countries that may be a driver of the change in tax rates during the COVID-19 era.

## Data Availability Statement

Publicly available datasets were analyzed in this study. This data can be found here: https://databank.worldbank.org/source/world-development-indicator; https://home.kpmg/it/it/home/services/tax/tax-tools-and-resources/tax-rates-online/corporate-tax-rates-table.html; https://worlduncertaintyindex.com/.

## Author Contributions

RL: writing and reviewing the manuscript. ZL: data collection and methodology. LG: writing the manuscript and estimations. All authors contributed to the article and approved the submitted version.

## Funding

We acknowledge the financial supports from the National Social Science Fund of China (Project#: 20BJL134).

## Conflict of Interest

The authors declare that the research was conducted in the absence of any commercial or financial relationships that could be construed as a potential conflict of interest.

## Publisher's Note

All claims expressed in this article are solely those of the authors and do not necessarily represent those of their affiliated organizations, or those of the publisher, the editors and the reviewers. Any product that may be evaluated in this article, or claim that may be made by its manufacturer, is not guaranteed or endorsed by the publisher.

## Footnotes

^1^Afghanistan, Albania, Algeria, Angola, Argentina, Armenia, Australia, Austria, Azerbaijan, Bangladesh, Belgium, Benin, Bolivia, Bosnia and Herzegovina, Botswana, Brazil, Bulgaria, Burkina Faso, Burundi, Cambodia, Cameroon, Canada, Chile, China, Colombia, Congo Republic, Congo DR, Costa Rica, Côte d'Ivoire, Croatia, Czech Republic, Denmark, Dominican Republic, Egypt, El Salvador, Ethiopia, Finland, France, Gabon, Georgia, Germany, Ghana, Greece, Guatemala, Honduras, Hungary, India, Indonesia, Iraq, Ireland, Israel, Italy, Jamaica, Japan, Jordan, Kazakhstan, Kenya, Korea Republic, Kuwait, Kyrgyz Republic, Latvia, Libya, Lithuania, Malaysia, Mexico, Moldova, Mongolia, Morocco, Mozambique, Myanmar, Namibia, Netherlands, New Zealand, Nicaragua, Nigeria, Norway, Pakistan, Panama, North Macedonia, Paraguay, Peru, Philippines, Poland, Portugal, Qatar, Romania, Russia, Rwanda, Saudi Arabia, Senegal, Sierra Leone, Singapore, Slovak Republic, Slovenia, South Africa, Spain, Sri Lanka, Sudan, Sweden, Switzerland, Thailand, Tunisia, Turkey, Uganda, Ukraine, the United Arab Emirates, the United Kingdom, the United States, Uruguay, Uzbekistan, Vietnam, Zambia, and Zimbabwe.
